# Bringing Top-End Endoscopy to Regional Australia: Hurdles and Benefits

**DOI:** 10.1155/2012/347202

**Published:** 2012-09-09

**Authors:** J. Van Den Bogaerde, D. Sorrentino

**Affiliations:** ^1^Department of Gastroenterology, Nambour General Hospital, Nambour, QLD 4560, Australia; ^2^University of Queensland and Sunshine Coast Clinical School, Nambour, QLD 4560, Australia; ^3^University of Udine Medical School, Udine 33100, Italy

## Abstract

This paper focuses on recent experience in setting up an endoscopy unit in a large regional hospital. The mix of endoscopy in three smaller hospitals, draining into the large hospital endoscopy unit, has enabled the authors to comment on practical and achievable steps towards creating best practice endoscopy in the regional setting. The challenges of using what is available from an infrastructural equipment and personnel setting are discussed. In a fast moving field such as endoscopy, new techniques have an important role to play, and some are indeed cost effective and have been shown to improve patient care. Some of the new techniques and technologies are easily applicable to smaller endoscopy units and can be easily integrated into the practice of working endoscopists. Cost effectiveness and patient care should always be the final arbiter of what is essential, as opposed to what is nice to have. Close cooperation between referral and peripheral centers should also guide these decisions.

## 1. Introduction

In Australia, it is probably easier to define what a teaching hospital is than a community hospital. A community hospital can be defined by what it is not, rather than what it is. It is not a tertiary referral center where research, training, and university affiliations are of primary importance. There are however many larger community hospitals in Australia where registrars rotate. Medical and nursing students are to be found even in small hospitals. A tertiary hospital refers more to the scope of hospital referral, so, for instance, a large hospital in Sydney to which several smaller hospitals send their difficult patients could rightly be called a tertiary hospital. There are however many large community hospitals which drain smaller hospitals, and they in turn would send patients that they could not manage to large university hospitals. 

The authors' hospital in Nambour, Queensland represents an interesting mix of practice. The endoscopy department conducts outreach endoscopy in 3 peripheral hospitals serving small towns of less than 30 000 people, upto 70 km from the base hospital. The base hospital has 400 beds and is a community hospital. We have very recently extended our endoscopy unit, transforming it from a single theatre complex-based room, into a standalone endoscopy unit, with dedicated endoscopy nursing staff and two large endoscopy suites. Within 5 years a new teaching hospital will be built, and the hospital district will become a tertiary center. These changes have enabled us to ponder on community-based endoscopy service in Australia, and on what is needed to upgrade to a teaching hospital endoscopic service.

Endoscopy is a fast moving field, where new techniques are constantly being instituted, resulting in better patient care and the need for less surgical intervention. Here we will review the major hurdles and the benefits in providing selected top end endoscopic service to a community hospital. The major hurdles to provision of excellent endoscopic services are infrastructure, personnel, and equipment.

## 2. The Hurdles

### 2.1. Infrastructure

Infrastructure is defined as the area in which endoscopy occurs, the patient flow to and from such area, as well as the recovery space. The majority of community hospitals in Australia use theatre complex space to perform endoscopy. In addition, surgeons with an interest in endoscopy perform most of their endoscopic procedures in this setting, often placing endoscopy patients on lists where other procedures are performed. This practice does not necessarily result in poor quality endoscopy, but dedicated endoscopists who perform a high volume of polypectomies in the hospital setting have the lowest rate of missed postcolonoscopy colorectal cancer [1]. 

In a nondedicated endoscopy room, storage of endoscopic equipment, training, and proficiency of endoscopy nurses, and patient turnover are not optimal. By definition, endoscopy is not a sterile procedure, and the culture of a sterile theatre environment and nursing protocols associated with it tend to clash with efficient endoscopic turnover. Sharing recovery space with postsurgical patients is often also a problem. Recovery protocols for postsurgical patients are not optimal for endoscopy patients where conscious sedation results in quick patient recovery. In addition, the use of CO_2_ insufflation results in easier recovery from colonoscopy [2, 3]. Recent experience in our center has emphasized the vast difference in attitude and efficiency when the theatre environment is exchanged for a dedicated endoscopy suite. Personnel morale and patient satisfaction are much higher in dedicated units.

Hence, the creation of dedicated endoscopy units with their own personnel is probably the first step which needs to be taken to improve endoscopic standards and implement new techniques [4, 5]. Patient numbers are obviously important as well, and a report from the surgical literature suggests that when the number of procedures exceeds 600 per year, a fully dedicated endoscopy room should be considered [6]. 

### 2.2. Personnel

There are no clear data regarding this, but a minimum of three dedicated endoscopists would be needed to justify the funding commitment of a dedicated unit. The techniques employed in the unit, would have to depend on the competence and training of the endoscopists. The ability to perform new techniques depends on having the right equipment, but more importantly having endoscopists with the confidence and training to perform these techniques safely. In general, new techniques are pioneered in teaching hospitals and often diffuse into the community setting when trainees become consultants in community hospitals. In small hospitals with stable staffing complements, this diffusion of knowledge does not occur quickly. 

In general, consultant endoscopists in smaller hospitals in Australia are senior and job mobility is minimal. Training of these individuals is difficult, since they are often clinically committed and cannot leave the hospital for long periods of time to master new techniques. Important teaching occurs at endoscopy forums, and new techniques such as endoscopic submucosal resection of large polyps [7] can be assimilated relatively easily by competent endoscopists, and gradually, confidence in removal of larger polyps can grow within the community endoscopy unit. However, other techniques are more difficult to master. Endoscopic Ultrasonography is a very good example [8]. In Australia competence in this technique is defined as doing 200 procedures under supervision, and 25 EUS-guided fine needle pancreas aspirations need to be undertaken according to American guidelines [9]. From a practical perspective this would be achievable in 6 to 12 months training but most senior clinicians cannot take 6 months off to train. Additionally there is competition for training positions, and many such procedures are allocated to training fellows. Training logistics represent an additional and very substantial hurdle.

### 2.3. Equipment

The equipment needed to provide acceptable endoscopy is closely related with the expertise and preference of the endoscopists. An optimal suite of equipment would include high definition and recent generation endoscopes, preferably with narrow band or equivalent image enhancement capabilities. However, somewhat counterintuitively, a recent meta-analysis failed to document significantly improved adenoma detection when standard and high definition colonoscopes were compared, emphasizing that endoscopic competence is probably more important than equipment [10].

With endoscope numbers, redundancy needs to be built in for unexpected events, such as sterilizer failure or scope breakage. Endoscopy lists are fluid, and urgent cases may be unexpectedly added, so a ready-to-go colonoscope and gastroscope should always be on standby. As shown in Table 1 equipment costs are substantial. Other items such as recovery equipment are not shown, but represent a substantial cost. 

## 3. The Benefits

This section will focus on what type of services a community endoscopy unit should provide, which services are “nice to have”, and which are rather left to teaching hospitals. There is no literature to guide these decisions, but enthusiasm and expertise of the endoscopic personnel and budgets are the determining factors. We will provide a guide for which technologies may fit into the community hospital setup and what the benefits are. 

### 3.1. Emergency Procedures

Emergency procedures are unplanned and often after hours, consisting mostly of upper gastrointestinal bleeds [11]. Community endoscopic units have been shown to be as effective and cost effective as academic centers [12], and accurate and early endoscopic diagnosis in the community setting improves outcomes in ICU patients with upper GI bleeding [13]. In patients with esophageal variceal bleeds, teaching hospitals were shown to adhere more closely to guidelines than community endoscopy units, but mortality did not differ significantly [14]. Similar data have been reported for nonvariceal upper GI bleeding [15].

Most competent endoscopists can apply clips and bands, use coagulation devices, and inject adrenaline to control bleeding. The extensive use of anticlotting agents has added a new dimension to upper and lower GI bleeding and the acute control of this. Mortality remains worryingly high particularly for variceal bleeds (15%) [11]. This will not be materially changed by new techniques, and reflects the frailty of the bleeding population. 

New additions to control bleeding include over-the-scope clip devices (OTSC) [16, 17] and nanopowder injection [18, 19]. These can be used with conventional endoscopy equipment, are easy to master, and could be useful in large and small endoscopy units. OTSC devices probably will find a role in treating colonic perforations [20–22], but could also be used in difficulty to control postpolypectomy bleeding. These techniques are not in general use in tertiary units yet and have yet to find their role in the community setting.

### 3.2. Elective Endoscopy

#### 3.2.1. Early Detection and Removal of Polyps in the Colon

No elective endoscopic task is more important than the early detection and removal of polyps in the colon. Institution of screening services such as FOB testing has been shown to increase endoscopy workload in community hospitals by 118% [23]. Surveillance of colonic polyps has been shown to reduce subsequent risk of colonic malignancy by upto 65% [24], but right-sided lesions are often missed [25]. Patients presenting with colorectal cancer 3 years after colonoscopy probably represent misdiagnosed lesions, with proximal missed lesions reported in 14.4% of patients [26]. Both left- and right-sided missed lesions have been reported in the community hospital-based setting [27]. Flat lesions are often missed and have been reported in almost 10% of patients undergoing screening colonoscopies, with a third of depressed lesions containing malignant cells [28]. A large community-based colonoscopy study reported a low adenoma detection rate (6.6%), and polypectomy follow-up recommendations that were not guideline based [29].

Endoscopy units, whether large or small, must achieve maximal detection of polyps, with particular focus on proximal lesions and particular care must be taken to find flat lesions and sessile serrated adenomas [30] which contribute to the colon cancer pathway [31, 32]. Serrated polyp detection rate is particularly challenging and does not necessarily mirror adenoma detection rate [33].

Retroflexion and third eye retroscopes have attempted to address polyps hidden in folds, particularly in the ascending colon. Cecal retroflexion is achievable in 94.4% of patients, with no reported complications in this series [34]. An additional 9.8% of adenomas were detected in the ascending colon. It is not clear whether a careful second pass down the ascending colon, without retroflexion, would have resulted in similar detection rates. The advantage of retroflexion is that it is safe, easy, and focuses the attention of the endoscopist on danger areas in the ascending colon. 

Similar results can be achieved with third eye retroscopes, although cost is an issue here. A recent report highlighted the success of this technology, showing an increased adenoma detection rate of 11%, and impressively a third more large (>10 mm) adenomas were discovered [35]. Somewhat surprisingly, larger polyps were easier to miss with conventional forward viewing. This observation has been confirmed by another group [36]. Withdrawal time is not substantially different between conventional and retroflexion cohorts [36]. Third eye technology is experimental and is not routinely used in tertiary centres, so the role of this in the community setting is not established.

Other techniques of increasing adenoma detection have focused on withdrawal time, but a recent report specifically addressing this issue has failed to show marked differences [37], and unsurprisingly other factors such as endoscopist skill and colon preparation appear to be more important [38, 39]. Increased experience of the nurse endoscopist assistant also increases adenoma detection [40]. It is probably sensible to audit endoscopists in units, large or small, and determine adenoma detection rates for each endoscopist. Although this sounds appealing, there are provisos to this. Is each endoscopist seeing the same patient cohort, and are lists directly comparable? The more experienced endoscopist may be doing more elderly and challenging patients, who would also probably have more pathology, thus creating the appearance of a higher adenoma detection rate. In addition, once a significantly lower detection rate is documented, how is this to be addressed? Must the low detector be removed from endoscopic duty, or retrained? The issues of quality improvement and auditing are complex, and are seen by some as naming and shaming of colleagues, adding to already substantial work stress [41].

Changing the appearance of the mucosa is now accepted as a method of increasing adenoma detection. Two methods exist: dye staining and equipment settings such as narrow band imaging. 

Dye stains increase precision of diagnosis in Barrett's esophagus [42] and ulcerative colitis [43]. Chromoendoscopy using indigo carmine increased adenoma detection rate in a large and well-designed study, from 36.3% to 46.2%, with a marginal increase in withdrawal times [44]. It is surprising that dye spray is not used more in busy community-based endoscopy units. Dye stains have the advantage of being cheap, but also dirty, time inefficient, and results are variable [45]. 

Narrow band imaging (NBI) uses filters to emphasize blue-coloured light, thus accentuating vascular structures and can accurately discriminate between hyperplastic and adenomatous polyps [46]. Dysplasia in ulcerative colitis [47] and early gastric cancer [48] are amenable to more precise analysis using NBI. In Barrett's mucosa, NBI has been shown to diagnose high-grade dysplasia with a very high sensitivity (96%) and specificity (94%) [49].

The concept of an optical biopsy, as opposed to a tissue biopsy is appealing [50]. In concert with high resolution and magnification endoscopy, precise and detailed evaluation of mucosa can be undertaken. 

The problem with NBI is that the field depth is much reduced when compared to white light. Inevitably white light is used to see an abnormality, and then interrogation with NBI assists in confirming the pathology. It certainly is a “nice to have” for endoscopists who use it regularly, and training endoscopists to use NBI is not daunting [51], but there is little evidence that NBI increased adenoma detection rate [52–55]. 

The next level of mucosal evaluation is confocal laser endomicroscopy, which uses laser to visualize cellular structures in the mucosa, producing real-time histology of suspicious lesions [56]. The most important application of this new technology is in evaluation of dysplasia in patients with long-standing ulcerative colitis [57] and Barrett's esophagus [58]. The role of these sophisticated imaging techniques is limited to large teaching hospitals. 

Endoscopist skill is therefore of greater importance than updating equipment in community hospitals.

#### 3.2.2. Small-Bowel Evaluation

Accurate diagnosis of small-bowel pathology is now achievable. Radiological techniques, particularly MR enterography, allow excellent visualization of the bowel, without the danger of radiation. The best evaluation of intraluminal pathology is capsule enteroscopy. Sensitivity and specificity are very high for any structural lesion of the small bowel [59, 60]. Setup costs are not daunting and this technology should be affordable even in small endoscopy units. Even experienced operators of these systems take time to complete a study, but the number of studies in community-based units should not exceed one or two per week. 

The problem with small-bowel evaluation comes when pathology is detected and intervention is necessary. A patient with iron deficiency anemia may have small-bowel vascular ectasia which is amenable to Argon plasma coagulation, or a polyp which can be removed endoscopically. There are three ways of accessing the small bowel: double balloon enteroscopy, single-balloon enteroscopy, and spiral enteroscopy. Double balloon is the established technology and achieves a higher completion rate and diagnostic yield than single balloon enteroscopy [61]. Spiral enterography is a new technique whereby an overtube with a distal thread is placed over a conventional colonoscopy and twisted into the small bowel [62]. The insertion time for spiral enterography appears to be less than double balloon, but depth of insertion is considerably less [63]. Stent insertion and therapeutic maneuvers may be easier with the spiral enterography due to overtube stabilization [64]. 

What role should these small-bowel techniques play in community endoscopy units? Capsule endoscopy would probably be the first choice, since it is easy, affordable, safe, and diagnostically accurate. If small-bowel pathology is diagnosed, it would be essential to perform therapeutic procedures, and the choice between the three systems may be challenging. Although double balloon is the more established technology, it is only available in the Fujinon system. It would therefore be expensive to acquire this if other endoscopy platforms are being used already. Olympus has a single-balloon system which probably would access most small-bowel pathology. If the capsule showed very distal disease, referral for double balloon could be undertaken, or single balloon attempted with recognition that failure and subsequent referral may occur. Spiral enteroscopy has the advantage of using different endoscopic platforms, but its role has not been sufficiently defined to make recommendations yet. More data using all three systems needs to be assessed before the role of each technology can be defined. 

#### 3.2.3. ERCP

Ironically the greatest advance in ERCP in the last 10 years is the development of MRCP, which has completely dispensed with the need for diagnostic ERCP. The absolute number of ERCP has thus come down dramatically, but interventional ERCP, and particularly removal of common bile duct stones in patients with cholangitis, is a life-saving procedure, which should be available in most large community-based hospitals. Techniques have not really changed in 10 years. The interplay between EUS and ERCP in challenging patients is an interesting development, but probably something for the large academic units with enthusiastic experts.

#### 3.2.4. EUS

Endoscopic ultrasound is an established technique, which is probably most useful in assessment and intervention of pancreaticobiliary disease. The difficulty in training consultants in this has been discussed. A recent publication from a large community hospital in Hong Kong documents an uptake of 2 to 3 patients per week, but importantly describes use of this technique for staging of esophageal and gastric cancer [65]. This would probably not represent the bulk of work in busy tertiary EUS units, and there is considerable doubt about whether there is enough work to justify the expense of this equipment in community hospitals. 

#### 3.2.5. CO_2_ Insufflation

One technical advance which has improved colonoscopy, both for patients and proceduralists, is CO_2_ insufflation. Although described more than 30 years ago [66], recent data have unequivocally shown superiority to room air insufflation [67]. Patient recovery and distention after the procedure is markedly reduced, and even the smallest unit should strive to change to CO_2_ insufflation. 

#### 3.2.6. Sedation

Nurse sedationists [68, 69] and proceduralist-driven sedation [70] are safe, acceptable, and cost effective. For routine endoscopy, this approach is acceptable. General practitioners trained in anaesthetics provide anesthetic service in many community endoscopy units [71]. Safety data from our region does not show any difference in outcomes between specialist and GP sedationists.

Unsedated colonoscopy is practiced in some community settings, but low patient acceptance rate (56.2%), and substantially lower completion rates would suggest that this is not an option in most community units [72].

## 4. Conclusions

In summary, the main hurdles in implementing an excellent endoscopic service in Australia are in our opinion budgetary constraints and personnel and infrastructural inadequacy. There is no doubt that the first and most crucial step to improving endoscopy is the creation of dedicated endoscopy units, preferably removed from the surgical theatre environment. There are also major bureaucratic obstacles to creating such a unit, and even if money is available, an enthusiastic team has to drive the process. Although recruiting and retaining dedicated luminal endoscopists are an important component to the success of this endeavor, the other foundation issues such as equipment, nursing staff, booking personnel, patient flow, and recovery protocols are more difficult to put into place. 

The techniques which could be easily introduced in community hospitals in western countries are latest generation endoscopes, newer clipping devices, retroflexion, chromoendoscopy, and small-bowel capsule endoscopy. Some methods of accessing the small bowel would be useful, although not essential. There are some relatively cheap modifications, which fit onto most endoscopes which would enable most units to perform small-bowel interventions. The endoscopic skills needed for this do not materially differ from those used in gastroscopy and colonoscopy, so training for these is not a major hurdle. 

Cost-benefit analysis and helpful discussion with hospital administrators are important in deciding what is absolutely essential to providing a top-class endoscopy service and what should be referred elsewhere. Emergency endoscopy procedures are obviously judged differently from more elective procedures. 

The final hurdle to good regional endoscopy lies in the endoscopist. There is no doubt that regional endoscopy services can be as good as large academic units [73], and there is no reason to suppose that an accomplished academic endoscopist in a tertiary unit is technically better than a busy and experienced community practitioner. Audit and training in the community setting may however be more difficult than in the tertiary setting and given high workloads and established practice. Integrating the work of full-time endoscopists in a newly created unit with established nonspecialist practitioners—who may be part time or surgically based endoscopists—can be achieved, but it needs to be done sensitively. As always, the personalities and abilities of medical and nursing role players need to be subservient to the needs of patient care.

## Figures and Tables

**Table 1:** Costs for an upgrade of an existing endoscopy unit.

Equipment	Cost (in AUD)
Endoscopy hanging cupboard	66 000
Image capturing system	35 000
Mobile image intensifier (ERCP)	250 000
Colonoscopes (5)	177 000
Gastroscopes (5)	160 000
Duodenoscopes (2)	74 000
Scope reprocessor unit (2)	130 000
Sterilizer	50 000
Patient trollies (12)	100 000
Ceiling pendants	300 000

Total	$1 342 000
